# Generational Differences: A Comparison of Weight-Related Cognitions and Behaviors of Generation X and Millennial Mothers of Preschool Children

**DOI:** 10.3390/ijerph16132431

**Published:** 2019-07-09

**Authors:** Ruiying Xiong, Kim Spaccarotella, Virginia Quick, Carol Byrd-Bredbenner

**Affiliations:** 1 School of Medicine, University of Pennsylvania, Blockley Hall, Philadelphia, PA 19104, USA; 2 Department of Biological Sciences, Kean University, 1000 Morris Avenue, Union, NJ 07082, USA; 3 Department of Nutritional Sciences, Rutgers University, 26 Nichol Avenue, New Brunswick, NJ 08901, USA

**Keywords:** mothers, generation x, millennials, weight, obesity

## Abstract

A ‘generation’ is an identifiable group sharing birth years and significant life events at critical developmental ages. There is a paucity of literature examining how parental cognitions and lifestyle behaviors differ by generation and whether generational differences are substantial enough to warrant consideration during the development of health interventions. This study compared generational differences in weight-related cognitions and lifestyle behaviors of mothers of young children who were categorized as Generation X (born 1965–1981, *n* = 158) and Generation Y (aka Millennials; born 1982–1999, *n* = 162). Survey results indicated that Generation X had significantly higher family affluence; thus, this was controlled in subsequent analyses. Analysis of covariance indicated that Millennials had more positive expectations about the benefits of engaging in healthy eating and physical activity than comparators, but not significantly so. Millennial mothers placed significantly higher value on physical activity for themselves than Generation X mothers, but both generations were neutral on the value of personal physical activity. No generational differences were noted in self-efficacy of mothers for promoting childhood obesity-prevention practices to children and self-efficacy for personally engaging in weight-protective behaviors. Millennial mothers had significantly more family meals/week, however generations did not differ on the value placed on family meals, where family meals were eaten, or whether media devices were used at mealtime. Few differences were noted between the generations for most child feeding behaviors, except that Millennials reported placing significantly less pressure on children to eat. Mothers’ modeling of weight-related behaviors as a means for children’s observational learning about healthy eating, physical activity, and sedentary behaviors did not differ by generational group. The eating behaviors of mothers differed little between generations. Millennial mothers allowed significantly more media devices in children’s bedrooms and personally engaged in more screen time daily than comparators. Overall, the two generational groups were more similar than different in weight-related cognitions as well as for personal and parenting lifestyle behaviors. The results suggest that tailoring interventions for individuals at a similar life-stage (e.g., mothers of young children) by generation may not be warranted.

## 1. Introduction

Obesity can be the result of genetic, behavioral, and/or environmental factors [1,2,3]. While genetic factors are not easy to modify, interventions targeting weight-related behaviors and the environment can be potential strategies to prevent excess weight gain of children [4,5,6,7]. According to Bandura’s Social Cognitive Theory, parents model and teach children positive behaviors [8]. For young children, parents serve as food gatekeepers and influence their eating habits, physical activities, sedentary behaviors, and beliefs about weight-related behaviors [9,10,11,12,13,14,15,16,17,18]. For instance, evidence has shown that maternal obesity is a strong predictor of childhood obesity [19]. Additionally, availability and accessibility of certain foods at home largely affects children’s food preferences and consumption patterns [9,14]. Parental awareness, and attitudes towards their own and their children’s physical activity levels also greatly influence children’s exercise levels [11], while home media device availability and parental rules on screen time significantly predict children’s time spent on sedentary behaviors [17]. Thus, interventions improving parental weight-related behaviors and home environments are essential for childhood obesity prevention.

Ideally, obesity prevention programs for preschoolers need to be multicomponent and family-based, because preschoolers spend a significant amount of time at home, and the home environment largely dictates their lifestyle [18,20,21]. Families are often the focus of health intervention programs because they provide a variety of components and opportunities for health behavior communication and maintenance [12].

Many childhood obesity prevention interventions and observational studies have examined parental characteristics [22,23,24,25,26,27]. However, limited research has considered how parent cognitions and lifestyle behaviors differ by generation and whether generational differences exist in weight-related parenting. A ‘generation’ is defined by Kupperschmidt [28] as an identifiable group that shares birth years and significant life events at critical developmental ages.

There are currently four adult generations in American society: The Silent Generation, the Baby Boomers, Generation X, and Millennials [29]. Two generations represent those who are currently parents of young children: Those born between 1982 and 2000 (commonly called Millennials or Generation Y) and those born between 1966 and 1981 (commonly labeled as Generation X). Given that these two generations grew up experiencing different environmental and life events, it is possible that their weight-related values and perspectives differ as well. For instance, Generation X witnessed a shift in communication tools whereas Millennials grew up surrounded by modern technology and the Internet [30]. Millennial students tend to value college education for its extrinsic or social capital benefits, such as for making more money or meeting new friends, whereas Generation X students tend to value intrinsic benefits, such as the appreciation of knowledge [31,32]. Millennials also are more individualistic, less altruistic at work [33,34], and experience greater job mobility than previous generations [35].

Common to both generations is the obesity epidemic, yet little research has examined whether Generation X and Millennials differ when it comes to their own personal health and weight-related behaviors. Given the importance of parents in creating home environments and lifestyles that affect child health and the emphasis marketing professionals place on generation differences, it is surprising that no research comparing generational differences in weight-related parenting cognitions and behaviors could be located [36,37].

Thus, the purpose of this exploratory study was to compare generational differences in weight-related cognitions and lifestyle behaviors of participants in the HomeStyles randomized controlled trial at baseline [38]. HomeStyles, a childhood obesity prevention program, aims to enable and motivate parents to shape their home environment and weight-related behaviors to prevent excessive weight gain in their preschool children. Understanding the differences between Generation X and Millennial parents could enable health educators to better design programs tailored to generational differences.

## 2. Materials and Methods

The study was approved by the Institutional Review Boards at Rutgers, The State University of New Jersey, and the University of Arizona. All participants gave informed consent. The study protocol [39] was reported in detail elsewhere and is described in brief below.

### 2.1. Sample

The sample was drawn from the final HomeStyles randomized control trial’s baseline analytic sample [40]. Multiple methods were used in the recruitment of this sample [41], including announcements distributed in person and posted online through various professional and community groups. These participants were aged between 20 and 45 years who had one or more 2 to 6-year-old child, were the family food gatekeeper, lived in study areas (New Jersey or Arizona), and had regular internet access. To control for possible confounding variables and increase sample homogeneity, additional eligibility criteria were applied a priori. Based on the concept that generational groups are a phenomenon unique to the prevailing culture and more applicable to “western” countries [42], participants were retained if they spoke English as their primary language and were born in the US. Additional eligibility criteria to maintain consistency between sex and family structure [43] were females living in dual-parent households. For this secondary data analysis, participants were further categorized based on age in Generation X (born 1965–1981) or Millennial (born 1982–1999) comparison groups. Additional details on the HomeStyles sample can be found elsewhere [40].

### 2.2. Instrument

Data were collected via an online survey comprised of an array of previously validated scales that were found to be reliable in the study population [38,44]. These scales are described in detail elsewhere [38,44] and are summarized below. These scales assessed parent health status, weight-related cognitions (i.e., values, outcome expectations, self-efficacy), and weight-related parenting behaviors and lifestyle behaviors. Table 1 reports the total items on each scale, answer choices, possible range of scores, and Cronbach’s alpha (when applicable). Parent health was assessed using the Centers for Disease Control and Prevention’s Health-Related Quality of Life (HRQOL) [45,46], depressive symptoms with the Patient Health Questionnaire-2 [47], and stress levels with Cohen’s Perceived Stress Scale [48].

Weight-related cognitions were assessed with five-point Likert scales. Values assessed included importance placed on family meals and physical activity for self [44]. Outcome expectations (belief that engaging in a particular behavior will lead to certain results) [49] for healthy eating and physical activity also were assessed. In addition, parent self-efficacy for promoting obesity-protective (healthy eating and weight management) behaviors in their children [44,50,51] as well as personally engaging in weight-protective behaviors [44] was assessed.

Weight-related parenting behaviors dealing with eating included weekly frequency of total family meals [52], family meals eaten at the dining room table, family meals eaten in front of the TV [53], and electronic devices that were used during family meals [54]. Measures of child feeding behaviors parents used were derived from an array of scales, including pressuring children to eat healthy foods [55], and/or restricting children’s eating of salty snacks and sweets [55], and using food to reward children for eating healthy foods [56,57]. Weight-related parenting behaviors related to physical activity included whether parents allowed electronic devices (e.g., TV, DVD, computers, tablets, phones, video games) in children’s bedrooms, the frequency in which parents engaged in active play with children, and the total time parents permitted children to spend on sedentary screen time [6]. Parent modeling of healthy weight-related behaviors focused on both eating [58,59,60] and physical activity [75].

The personal eating behaviors of parents included restraint, disinhibited, and emotional eating, evaluated with the Three-Factor Eating Questionnaire [61,62,63], intake of fruits/vegetables and calories from fat using Block screeners [64,65,66,67], and intake of sugar-sweetened beverages [68,69]. Other personal weight-related behavior measures were the modified streamlined International Physical Activity Questionnaire, used to assess physical activity level [70,71], sedentary screen-time [72], and the usual daily sleep duration from the Pittsburgh Sleep Quality Index [73,74].

Participants also reported demographic data, including age, race, education level, and number of children under 18 years living in their household, as well as the age and sex of one randomly selected young child in the household. The 4-item family affluence scale was used as a proxy for family socioeconomic status [76,77].

### 2.3. Data Analysis

Descriptive analyses (e.g., means, standard errors) were performed to assess the demographic and socioeconomic characteristics of participants and their children at baseline. Independent t-tests to examine demographic and socioeconomic characteristic differences between Generation X and Millennial mothers were conducted. Spearman rank order correlations were performed to determine significant correlations among socioeconomic characteristics and generation type. The internal consistency using Cronbach’s alpha of all parent weight-related behaviors and cognitions were assessed to examine reliability. An analysis of covariance controlling for family affluence was conducted to examine generational differences of parent weight-related behaviors and cognitions between Generation X and Millennial mothers of preschool children. To detect a medium effect size (*f* = 0.25) between the two groups of mothers, with 95% power at the 5% significance level, a minimum sample size of *n* = 210 participants was needed. Power was calculated using G*Power (version 3.1; Heinrich Heine University Dusseldorf, Germany). To determine effect sizes, Cohen’s *d* was conducted on variables that were found to be significantly (*p* < 0.05) different between groups of mothers. All analyses were performed in SPSS, version 24 (Chicago, IL, USA).

## 3. Results

**Figure 1:**
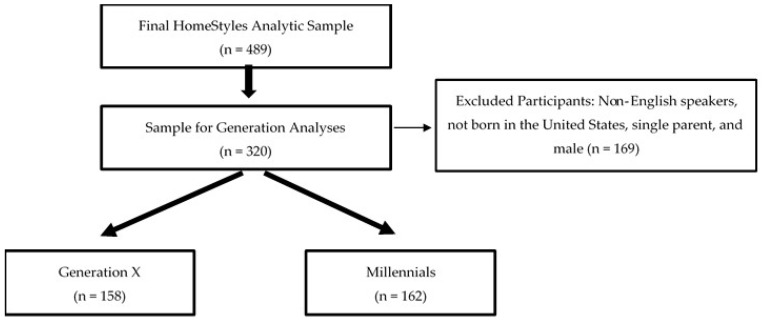
Analytic sample of the HomeStyles randomized control trial participants for analyses by generation.

Figure 1 depicts the study sample drawn from the final analytic sample of the HomeStyles randomized controlled trial at baseline [40]. Of the 489 participants, the sample was further narrowed by applying eligibility criteria, resulting in a sample of 320 mothers (*n* = 158 Generation X and *n* = 162 Millennials).

The mean age was 36.97 ± 3.01 SD years for mothers in Generation X and, as expected, was significantly (*t* = 24.83, *df* = 318, *p* < 0.001) higher than the mean age (28.39 ± 3.17SD years) of Millennial mothers. Most mothers (68%) classified themselves as White. Significantly (*p* < 0.001) more (73%) Generation X mothers had at least a bachelor’s degree or higher than Millennial mothers (38%). Similarly, significantly (*p* < 0.001) more Generation X mothers (74%) were employed compared to Millennial mothers (53%). Most participants had one (27.5%) or two children (45%) with no significant differences in the number of children between Generation X or Millennial mothers. Additionally, the children that parents reported on had a mean age of 3.83 ± 1.05 SD years old, with about half being boys (51%). There were no significant child sex differences between Generation X or Millennial mothers; however, children from Generation X moms were slightly older (4.06 ± 1.05 SD years) than those of Millennial moms (3.61 ± 1.01 SD years). The mean family affluence score for both groups was moderate, with Generation X mothers having significantly higher affluence on this 0 to 9-point scale than Millennial mothers (i.e., 6.11 ± 1.55SD vs 4.96 ± 1.74SD, *p* < 0.001). Education level, employment, and family affluence scores were highly and significantly correlated with each other (r ≥ 0.37, *p* < 0.001). To avoid collinearity, only family affluence was used as a covariate in subsequent analyses as this variable had the highest correlation (*r* = 0.36, *p* < 0.001) with generational differences of mothers.

As shown in Table 1, mothers in this study had a moderate health status, good control of stress, and had low levels of depressive symptoms. Thus, health measures did not differ between the generations. Regarding weight-related cognitions, mothers placed high value on family meals as well as healthy eating and physical activity outcome expectations. Although Millennials had more positive expectations about the benefits of engaging in healthy eating and physical activity than comparators, these differences did not reach the threshold for significance. Overall, mothers had moderate self-efficacy scores for promoting childhood obesity-protective behaviors and personally engaging in weight-protective behaviors. Millennial mothers placed significantly higher value on physical activity for themselves than Generation X mothers (Cohen’s *d* = 0.20), but both were neutral on the value of personal physical activity. Generational differences in the self-efficacy of mothers for promoting childhood obesity-prevention practices to children, as well as self-efficacy for personally engaging in weight-protective lifestyle behaviors, were not apparent.

Both generations reported frequent family meals that were mostly eaten at a dining table. Millennial mothers reported significantly more family meals per week (Cohen’s *d* = 0.20); however generations did not differ in the value placed on family meals or where family meals were eaten or whether media devices were used at mealtime. Few differences were noted between the generations in most child feeding behaviors, except Millennials reported placing significantly less pressure on children to eat (Cohen’s *d* = 0.24). Both Millennial and Generation X mothers permitted media device use in children’s bedrooms, with Millennial mothers allowing significantly more media devices in children’s bedrooms (Cohen’s *d* = 0.31). Mothers were neutral about the value of modeling healthy behavior to children and did so only about half the days in a week. Mothers’ modeling of weight-related behaviors as a means for children’s observational learning about healthy eating, physical activity, and sedentary behaviors did not differ by generational group.

The eating behaviors of mothers differed little between generations. Most engaged in little restraint, disinhibited, or emotional eating, although Millennial mothers engaged in non-significantly more emotional eating. Millennial mothers also had more sugar-sweetened beverages than Generation X mothers (approached significance at *p* = 0.058, Cohen’s *d* = 0.39). Both groups had fruit and vegetable intakes lower than recommended (five or more per day) [78] and fat intake as a percentage of total calories higher than recommended (less than 35%) [79]. Physical activity was low among both generations. Daily screen time exceeded five hours and was significantly higher for Millennial mothers (Cohen’s *d* = 0.33). Sleep duration barely met the minimum recommendation of seven hours/day [80].

## 4. Discussion

Previous research has shown generational differences in career patterns [35] and expectations from education [31,81], but few studies have examined how parents from differing generations (i.e., Generation X vs Millennials) who both have children of the same age group (i.e., preschoolers) differ. The findings of this study reveal that Generation X mothers were more educated than Millennial mothers, which is not surprising as Millennial mothers are younger and might continue their education at a later age for a workplace advancement or salary increase [32]. Additionally, Generation X parents had higher family affluence scores, likely because they had more years in the workplace and more education, which are associated with higher paying jobs. Conversely, the lower employment rate of Millennial mothers may have provided more time for meal preparation and may explain why Millennial parents had more weekly family meals than Generation X parents.

Generation X mothers were more likely, though not significantly, to engage in emotional eating. The concept of emotional eating proposes that individuals tend to cope with dysphoric mood through eating [62] and that those who are emotional eaters may have an increased likelihood of being overweight or having weight-related problems. Others have reported that emotional eating is linked more closely to stress [82]; although stress did not differ significantly between generations in the present study, research has suggested that workplace stress can lead to overeating [83]. Perhaps greater hours of employment and associated workplace stress experienced by Generation X mothers influenced eating patterns differently than the stressors at home experienced by Millennial mothers.

Millennial mothers consumed more servings of sugar-sweetened beverages daily when compared to Generation X mothers. This small effect size difference may be linked to the lower affluence of Generation X mothers [84]. Millennial mothers also spent more time watching television or using other sedentary media devices than Generation X mothers. In addition, millennial parents also reported that more media devices were present in children’s bedrooms. However, these effects sizes were small. As a generation growing up with the popularization of technology and media devices [30], Millennials are used to living in a media-surrounded environment and appear to be passing this on to their children, which could increase sedentary time with media devices and risk of obesity [85].

The commonly held idea that older mothers tend to have more confidence in their parenting skills was not supported in this study and other research [86]. Indeed, both generations only somewhat agreed they were confident in promoting childhood obesity-protective practices. In addition, the findings indicate several areas where mothers of preschoolers might benefit from nutrition education opportunities. For instance, parents engaged in limited modeling of healthy behaviors to children had dietary intakes incongruent with dietary recommendations (e.g., high percent of total calories from fat), permitted practices associated with less healthy food intake at mealtimes (e.g., eating in front of the TV, media device use at mealtime [87,88,89,90]), and did not highly value physical activity for themselves.

When considering the study findings, it is important to differentiate between observed differences that are truly generational and those that could be explained by age. The present study compared two populations experiencing the same life events (raising a preschooler) in different life stages. Hence, it was difficult to discern the effects of maturity apart from generation. In addition, the sharp demarcation of years used to categorize participants born might cause bias. For instance, participants born in 1982 were categorized as Generation X while those born in 1981 were assigned to the Millennial generation. It is likely that those born close to these cut off years share the characteristics of both generations. Although this study was focused on weight-related behaviors, it was not possible to control maternal body weight because this data point was not included in the baseline survey. However, given that women’s weight tends to increase with age [78], coupled with the general lack of generational differences observed, suggests that maternal weight was unrelated to findings. The sample was a convenience sample and not necessarily representative of the population of mothers of preschoolers, thus findings should be viewed with caution. Additionally, the multiple tests of significance may increase the risk of a type 1 error. Additionally, the effect sizes for variables that were significantly different between Generation X and Millennial mothers was small. Future studies with a larger and more diverse sample of mothers are needed to confirm our exploratory study findings.

Even with these limitations, the study has many strengths. The sample of participants was demographically diverse, so the results can be applied to other health education programs to better develop tailored interventions. A comprehensive array of variables measured with valid, reliable scales allowed identification of possible confounders, and a homogeneous sample enabled a generational comparison. To our knowledge, this is the first study evaluating generational differences in weight-related behaviors and food-related parenting styles.

## 5. Conclusions

Despite the emphasis marketers place on generational differences when aiming to change purchasing patterns [36,37], this study revealed that generational groups were more similar than different in weight-related cognitions as well as personal lifestyle and parenting behaviors. Similarly, other well-designed generational studies on topics ranging from work-related attitudes and values [91,92,93] to efficacy of learning technologies and instructional design [94], run counter to the notions of marketers that Millennials differ significantly from the previous generation. Findings led researchers to conclude that differences attributable to the generation are “essentially zero” [91] (p. 375) and there is “little solid empirical evidence supporting generationally based differences” [95] (p. 308), thus because “meaningful differences between generations probably do not exist” [91] (p. 375), “targeted organizational interventions addressing generational differences may not be effective” [92] and generational differences are “not salient enough to warrant the specification of different instructional designs or the use of different learning technologies” [94] (p21). Likewise, the findings of the study reported here suggest that the effort and expense of tailoring nutrition and health interventions for individuals at a similar life-stage (e.g., parenting young children) by generation may not be indicated. However, this conclusion warrants further investigation to confirm the findings. Additionally, results from this study indicate that parents of preschoolers, regardless of their generation, could benefit from nutrition education.

## Figures and Tables

**Table 1:** Comparison of weight-related cognitions and behaviors between Generation X (born 1965 to 1981) and Millennial (born 1982 to 1999) mothers (*n* = 320).

Measures	# of Items	Scale Type	Possible Score Range	Cronbach’s Alpha	Generation X Mean ± SE (*n* = 158)	Millennials Mean ± SE (*n* = 162)	F ^†^	*p*-Value
Health								
Control of Stress [48]	2	4-point frequency rating ^A^	1–4	0.77	3.37 ± 0.06	3.41 ± 0.06	0.13	0.720
Health Status [45,46]	1	5-point excellence rating ^B^	1–5	*	3.40 ± 0.08	3.44 ± 0.08	0.13	0.723
Depression [47]	2	4-point frequency rating ^A^	1–4	0.80	1.51 ± 0.06	1.56 ± 0.06	0.44	0.507
Weight-Related Cognitions								
Value Placed on Family Meals [44]	3	5-point agreement rating ^C^	1–5	0.59	4.41 ± 0.05	4.50 ± 0.05	1.34	0.249
Value Placed on Physical Activity for the Self [44]	2	5-point agreement rating ^C^	1–5	0.88	2.73 ± 0.09	2.99 ± 0.09	3.90	0.049
Healthy Eating Outcome Expectations [44]	6	5-point agreement rating ^C^	1–5	0.92	4.51 ± 0.04	4.63 ± 0.04	3.43	0.065
Physical Activity Outcome Expectations [44]	6	5-point agreement rating ^C^	1–5	0.93	4.39 ± 0.05	4.52 ± 0.05	3.28	0.071
Self-Efficacy for Promoting Childhood Obesity-Protective Practices [44,50,51]	12	5-point confidence rating ^D^	1–5	0.86	3.72 ± 0.06	3.77 ± 0.05	0.43	0.510
Self-Efficacy for Personally Engaging in Weight-Protective Behaviors [44]	5	5-point confidence rating ^D^	1–5	0.82	3.15 ± 0.07	3.32 ± 0.07	2.55	0.112
Weight-Related Parenting Behaviors								
Family Meal frequency/week [52]	3	0–7 days for breakfast, lunch, dinner summed	0–21	*	12.30 ± 0.39	13.41 ± 0.38	4.02	0.046
*Family Meal Location* [53]								
At Dining Table (days/week)	1	0–7 days	0–7	*	5.12 ± 0.19	4.82 ± 0.18	1.18	0.278
In Front of TV (days/week)	1	0–7 days	0–7	*	1.96 ± 0.20	2.08 ± 0.20	0.16	0.691
Media Device Use at Family Meals [54] (days/week)	1	0–7 days	0–7	*	1.45 ± 0.19	1.66 ± 0.19	0.55	0.461
Pressures Child to Eat [55]	3	5-point agreement rating ^C^	1–5	0.64	2.35 ± 0.07	2.11 ± 0.07	5.09	0.025
Rewards Child with Food [55]	3	5-point frequency rating ^E^		0.74	2.33 ± 0.06	2.38 ± 0.06	0.33	0.566
Restricts Child Intake of Salty Snacks & Sweets [56,57]	2	5-point agreement rating ^C^	1–5	0.56	3.74 ± 0.07	3.70 ± 0.07	0.17	0.682
Media Device Allowed in Child Bedrooms [6]	5	yes/no ^F^	0–5	*	0.93 ± 0.12	1.32 ± 0.12	4.77	0.030
Playing Actively with Children (days/week) [6]	2	8-point modeling scale ^G^	0–7	0.62	3.83 ± 0.15	3.59 ± 0.15	1.25	0.264
Time Children are Allowed to Use Sedentary Media Devices [6] (min/day)	1	minutes/day	0–1440	*	470.76 ± 60.36	496.05 ± 59.57	0.84	0.772
Parent Modeling of Healthy Eating [58,59,60]	4	5-point agreement rating ^C^	1–5	0.74	3.65 ± 0.07	3.53 ± 0.06	1.56	0.213
Parent Modeling of Physical Activity (days/week) [6]	2	8-point modeling scale ^G^	0–7	0.53	3.31 ± 0.11	3.15 ± 0.10	1.08	0.300
Parent Modeling of Sedentary Activity (days/week) [6]	2	8-point modeling scale ^G^	0–7	0.72	3.88 ± 0.08	3.76 ± 0.08	1.09	0.297
Weight-Related Lifestyle Behaviors								
Dietary Restraint [61,62,63]	4	4-point true/false scale ^H^	1–4	0.71	2.39 ± 0.06	2.38 ± 0.06	0.01	0.930
Disinhibited Eating [61,62,63]	3	4-point true/false scale ^H^	1–4	0.75	2.05 ± 0.06	2.05 ± 0.06	0.01	0.988
Emotional Eating [61,62,63]	3	4-point true/false scale ^H^	1–4	0.89	2.29 ± 0.08	2.10 ± 0.08	2.97	0.086
Fruit/Vegetable (serv/day) [64,65,66,67]	7	6-point servings scale ^I^	0–12.17	*	4.31 ± 0.14	4.18 ± 0.14	0.45	0.503
% Total Calories from Fat [64,65,66,67]	17	5-point servings scale ^J^	0–100	*	36.42 ± 0.47	36.71 ± 0.47	0.18	0.672
Sugar-Sweetened Beverages [68,69] (serv/day)	4	9-point servings scale ^K^	0–4.6	*	0.58 ± 0.06	0.74 ± 0.06	3.62	0.058
Physical Activity Level [70,71]	3	8-point exercise scale ^L^	0–42	*	13.25 ± 0.78	13.62 ± 0.77	0.11	0.746
Screen time [72] (minutes/day)	1	minutes/day	0–1440	*	307.63 ± 22.15	385.90 ± 21.86	5.98	0.015
Sleep Duration [73,74] (hours/day)	1	hours/day	0–24	*	7.04 ± 0.10	7.03 ± 0.10	0.01	0.944

* Not applicable, † analysis of covariance controlling from family affluence, ^A^ 4-point frequency rating: Not at all, several days, more than half the days, nearly every day; scored 1 to 4, respectively; higher score indicates greater frequency. ^B^ 5-point excellence rating: Poor, fair, good, very good, excellent; scored 1 to 5, respectively; higher score indicates better health. ^C^ 5-point agreement rating: Strongly disagree, disagree, neither agree nor disagree, agree, strongly agree; scored 1 to 5, respectively, with scoring reversed for negatively-worded statements; the scale score equals the average of item scores; higher scale scores indicate greater expression of the trait. ^D^ 5-point confidence rating: Not at all confident, not confident, confident, quite confident, very confident; scored 1 to 5, respectively; higher scale scores indicate greater confidence. ^E^ 5-point frequency: Never, rarely, sometimes, most of the time, always; scored 1 to 5, respectively; higher scores indicate greater frequency. ^F^ Response for total type of media devices (TV, DVD player, computer/laptop, smart phone/tablet/leap pad, video games) in child’s bedroom. ^G^ 8-point modeling days/week: 0 (almost never), 1, 2, 3, 4, 5, 6, and 7; days averaged to create the scale score; higher scores indicate more frequent modeling. ^H^ 4-point true/false scale: Definitely false, mostly false, mostly true, definitely true; higher scores indicate it is true. ^I^ 6-point servings rating: <1/week, 1/week, 2 to 3 times/week, 4 to 6 times/week, 1/day, >1/day scale scoring algorithm is protected by copyright and described in detail elsewhere [64]; higher scores indicate greater intake. ^J^ 5-point servings rating: 1 time/month or less, 2 to 3 times/month, 1 to 2 times/week, 3 to 4 times/week, 5 or more times/week; scored 0 to 4, respectively; the scale scoring algorithm is protected by copyright and described in detail elsewhere [64]; higher scores indicate greater intake. ^K^ 9-point servings rating: <1 time/week, 1 day/week, 2 days/week, 3 days/week, 4 days/week, 5 days/week, 6 days/week, 7 days/week, >1 time/day; scored 0 to 8, respectively; higher scores indicate greater frequency. ^L^ 8-point exercise days/week: 0, 1, 2, 3, 4, 5, 6, and 7; days/week weighted by exercise intensity (weights of 1, 2, 3 for walking, moderate, and vigorous activity, respectively) and summed to create the scale score; higher scale scores indicate greater activity level.
